# Electronic properties of (Sb;Bi)_2_Te_3_ colloidal heterostructured nanoplates down to the single particle level

**DOI:** 10.1038/s41598-017-09903-w

**Published:** 2017-08-29

**Authors:** Wasim J. Mir, Alexandre Assouline, Clément Livache, Bertille Martinez, Nicolas Goubet, Xiang Zhen Xu, Gilles Patriarche, Sandrine Ithurria, Hervé Aubin, Emmanuel Lhuillier

**Affiliations:** 1 Sorbonne Universités, UPMC Univ. Paris 06, CNRS-UMR 7588, Institut des NanoSciences de Paris, 4 place Jussieu, 75005 Paris, France; 2 Department of Chemistry, Indian Institute of Science Education and Research (IISER), Pune, 411008 India; 3 Laboratoire de Physique et d’Étude des Matériaux, PSL Research University, CNRS UMR 8213, Sorbonne Universités UPMC Univ Paris 06, ESPCI ParisTech, 10 rue Vauquelin, 75005 Paris, France; 4 Laboratoire de Photonique et de Nanostructures (CNRS- LPN), Route de Nozay, 91460 Marcoussis, France

## Abstract

We investigate the potential use of colloidal nanoplates of Sb_2_Te_3_ by conducting transport on single particle with in mind their potential use as 3D topological insulator material. We develop a synthetic procedure for the growth of plates with large lateral extension and probe their infrared optical and transport properties. These two properties are used as probe for the determination of the bulk carrier density and agree on a value in the 2–3 × 10^19^ cm^−3^ range. Such value is compatible with the metallic side of the Mott criterion which is also confirmed by the weak thermal dependence of the conductance. By investigating the transport at the single particle level we demonstrate that the hole mobility in this system is around 40 cm^2^V^−1^s^−1^. For the bulk material mixing n-type Bi_2_Te_3_ with the p-type Sb_2_Te_3_ has been a successful way to control the carrier density. Here we apply this approach to the case of colloidally obtained nanoplates by growing a core-shell heterostructure of Sb_2_Te_3_/Bi_2_Te_3_ and demonstrates a reduction of the carrier density by a factor 2.5.

## Introduction

Bismuth and antimony chalcogenides (tetradymite group with formula such as (Sb;Bi)_2_(Se;Te)_3_) have attracted great interest in the past for their thermoelectric properties^1–5^. The heavy mass of these materials leads to a large spin orbit coupling which results in an inverted band structure. Over this past decade, it is the original electronic structure of bismuth and antimony chalcogenides which has driven most of interest in these compounds. Indeed they appear as model 3D topological insulator^6–9^, with conducting surface-states and an insulating core, as long as the material can be obtained under an intrinsic form.

Sb_2_Te_3_ is a 0.3 eV band gap semiconductor. This material has common antisite defects^10–12^ where Sb atoms replace Te atoms, which tends to result in a p-type doping^13^. Controlling the bulk carrier density in topological insulator compounds is a key challenge since the Fermi level of the material needs to be close to its Dirac point for electronic transport to be dominated by topologically protected surface states. Moreover, the conductance of the material is the sum of the surface and bulk contribution. Because of the narrow band gap nature of these topological insulator materials and their deviation from stoichiometry, the bulk is generally not as insulating as desired. By reducing the bulk carrier density and the associated conductance, the weight of the surface contribution in transport is expected to increase and make the surface observation more likely to occur.

The Mott criterion can be used to estimate whether the material will behave as a metal or as an insulator. Metallic behavior is expected to occur if ${a}_{0}{n}^{1/3} > 0.25$ ^14^, where *n* is the carrier density and ${a}_{0}=\frac{{h}^{2}{\varepsilon }_{0}{\varepsilon }_{r}}{\pi {m}^{\ast }{e}^{2}}$ the Bohr radius with *h* the Planck constant, ${\varepsilon }_{0}$ the vacuum permitivity, ${\varepsilon }_{r}$ the material dielectric constant, ${m}^{\ast }$ the effective mass and *e* the proton charge. Due to large dielectric constant (ε > 50) of Sb_2_Te_3_, the Bohr radius is large which makes the Mott criterion fulfilled even for low carrier densities. Typically the threshold carrier density is estimated to be ≈2 × 10^16^ cm^−3^ assuming an effective mass of 0.1 m_0_ where m_o_ is rest mass of an electron. As a result, Sb_2_Te_3_ typically behaves as a metal^6^. Alloying n-type (Bi_2_Se_3_ and Bi_2_Te_3_) and p-type (Sb_2_Te_3_) materials is a possible way to obtain a charge compensation and reduce the overall bulk carrier density. While this type of approaches have been extensively studied for bulk and thin film materials^15–17^ almost no work has been dedicated to colloidally synthesized materials.

The tetradymites are layered 2D materials^18, 19^. Each layer is 1 nm thick and is composed of 5 atoms (quintuplet). So far, most of the efforts towards growth of these materials have been focused on physical methods such as molecular beam epitaxy^20^, chemical vapor deposition^21^, pulsed laser deposition and exfoliation^22^. Chemical solvothermal methods have been proposed^23, 24^, however all these works were driven by the investigation of the thermoelectric properties and very little work was dedicated to the understanding of the electronic and spectroscopic properties of (Sb;Bi)_2_Te_3_ obtained under colloidal form. In this letter, we develop a colloidal synthesis of Sb_2_Te_3_ nanoplates and investigate their transport properties from a thin film down to the single particle level. We demonstrate that the material is indeed *p*-type. In the last part of the paper, we focus on the control of the carrier density within these plates by growing in solution a heterostructure combining an *n*-type (Bi based) and a *p*-type (Sb based) layer. We obtain by this way a decrease of the bulk carrier density by a factor ≈ 3. This work paves the way for the use of colloidal heterostructure as model 3D topological insulator.

## Results and Discussion

### Synthesis

The chemical synthesis of Sb_2_Te_3_ nanoplates has been investigated using solvothermal methods in aqueous^25–28^, or polar organic solvents^29–31^. We see two main limitations to these approaches, which are (*i*) the risk of oxidation of the material, and (*ii*) the final thickness of the nanoplate being limited to thick sheets (>50 nm^26, 27^). Hot injection methods in organic solvents are well established and lead to high monodispersity. Synthesis of Sb_2_Te_3_ nanoparticles in organic medium has also been proposed^32^. The latter is based on the thermal decomposition of single precursor containing antimony and tellurium at high temperature^33–35^. In this report, we rather use a bulky antimony precursor of antimony oleate, prepared from antimony acetate in presence of excess of oleic acid at a temperature where the acetic acid can be removed under vacuum (85 °C).

In a typical synthesis, the temperature is raised around 200 °C under Ar and the Te precursor (trioctylphosphine complexed with Te) is quickly injected in the flask. The reaction is conducted in non-coordinating solvent such as octadecene since we observe that coordinating solvent such as oleylamine leads to the formation of oxide instead of the telluride. The solution rapidly darkens, and after 1 min a grey metallic appearance is observed. The product is cleaned via addition of polar solvent and by the help of centrifugation. The particles can be stored in non-polar solvents such as hexane and toluene, but typically an immediate precipitation of the suspension is observed.

1 $$Sb{(OAc)}_{3}+OA\mathop{\mathop{\longrightarrow }\limits^{{80}^{\circ }C}}\limits_{vacuum}Sb{(OA)}_{3}+TOPTe\mathop{\mathop{\longrightarrow }\limits^{{200}^{\circ }C}}\limits_{Ar}S{b}_{2}T{e}_{3}$$

**Figure 1:**
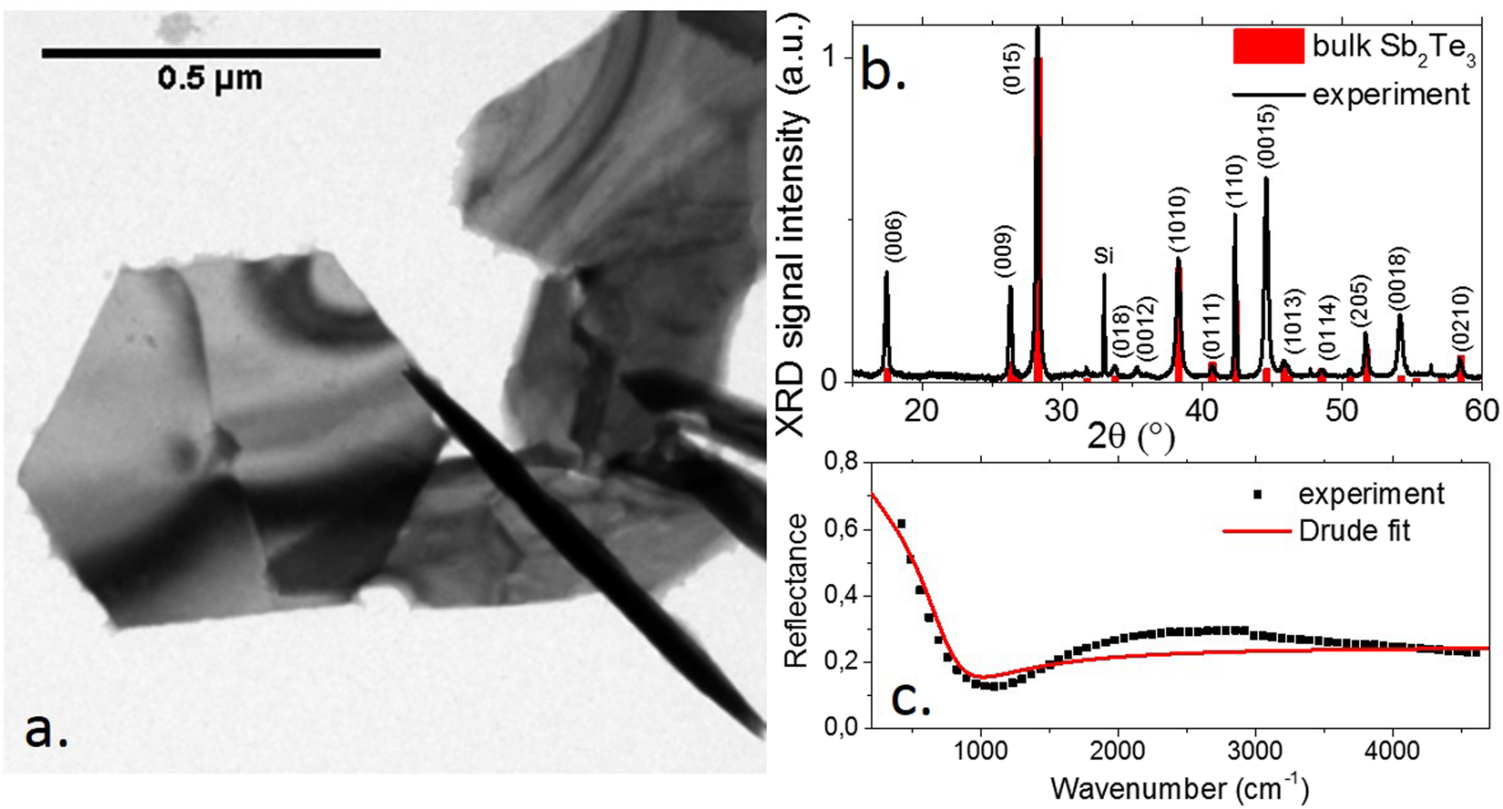
(**a**) TEM image of Sb_2_Te_3_ nanoplates. (**b**) X-ray diffraction pattern for a film of Sb_2_Te_3_ compared with reference. (**c**) Reflectance spectrum of a film of Sb_2_Te_3_ nanoplates and its empirical fit.

The obtained nanoplates typically present a hexagonal structure with lateral size ranging from 200 nm to 1 µm and a thickness from a few quintuplets (QL) up to 50 nm, see Figs 1a and S7. The detailed investigation of the effects of temperature, synthesis duration and stoichiometry on the final product is discussed in the SI, see Figs S1 to S5. The diffraction peaks from the XRD pattern are fully consistent with the trigonal phase (R $\bar{3}$ m) of Sb_2_Te_3_ (00-015-0874), see Fig. 1b. Energy dispersive X-ray (EDX) analysis (Fig. S6 and Table S1) confirmed the presence of both antimony and tellurium in the final compound and showed that the material is very close to stoichiometry Sb_2_Te_3+x_ with x = −0.1 ± 0.05, but is systematically Te deficient, consistent with previous report of this material^10–12^. This non stoichiometry of the compound is responsible for the metallic aspect of the solution and is further confirmed by the reflectance measurement, see Fig. 1c. The IR spectrum in Fig. 1c is poorly structured which suggests that the absorption results from free electrons.

To confirm this hypothesis, we can model the reflectivity assuming a Drude model^36, 37^ for the free electrons. In this case, the expression of the real $({\varepsilon }_{1})$ and imaginary $({\varepsilon }_{2})$ part of the dielectric constant are given by

2 $${\varepsilon }_{1}(\omega )={\varepsilon }_{\infty }-\frac{{\omega }_{p}^{2}}{{\omega }^{2}+{\gamma }^{2}}$$

and

3 $${\varepsilon }_{2}(\omega )=\frac{\gamma {\omega }_{p}^{2}}{\omega ({\omega }^{2}+{\gamma }^{2})}$$

where ${\varepsilon }_{\infty }$ is the dielectric constant at high frequency, ${\omega }_{p}$ the plasmon frequency and γ the damping rate. The reflectivity signal is given for a semi-infinite medium, by

4 $$R(\omega )=\frac{{(n-1)}^{2}+{k}^{2}}{{(n+1)}^{2}+{k}^{2}}$$

with *n* and *k* respectively the real and imaginary part of the optical index. The latter can be related to the dielectric constant by

5 $$n(\omega )=\sqrt{\frac{{\varepsilon }_{1}+\sqrt{{\varepsilon }_{1}^{2}+{\varepsilon }_{2}^{2}}}{2}}$$

and

6 $$k(\omega )=\sqrt{\frac{\sqrt{{\varepsilon }_{1}^{2}+{\varepsilon }_{2}^{2}}-{\varepsilon }_{1}}{2}}.$$

We obtain a reasonable agreement with obtained experimental data, see Fig. 1c, assuming ${\omega }_{p}=2460\,c{m}^{-1}$ and $1/\gamma =14fs$. From the plasmon frequency, we can estimate the carrier density *n* from the equation

7 $$\hslash {\omega }_{p}=\sqrt{\frac{n{e}^{2}}{{\varepsilon }_{\infty }{m}^{\ast }}}$$

to be $n=3.6\times {10}^{19}c{m}^{-3}$ which is consistent with the realization of the Mott criterion of metallic nature.

### Transport in Sb_2_Te_3_ nanoplates

**Figure 2:**
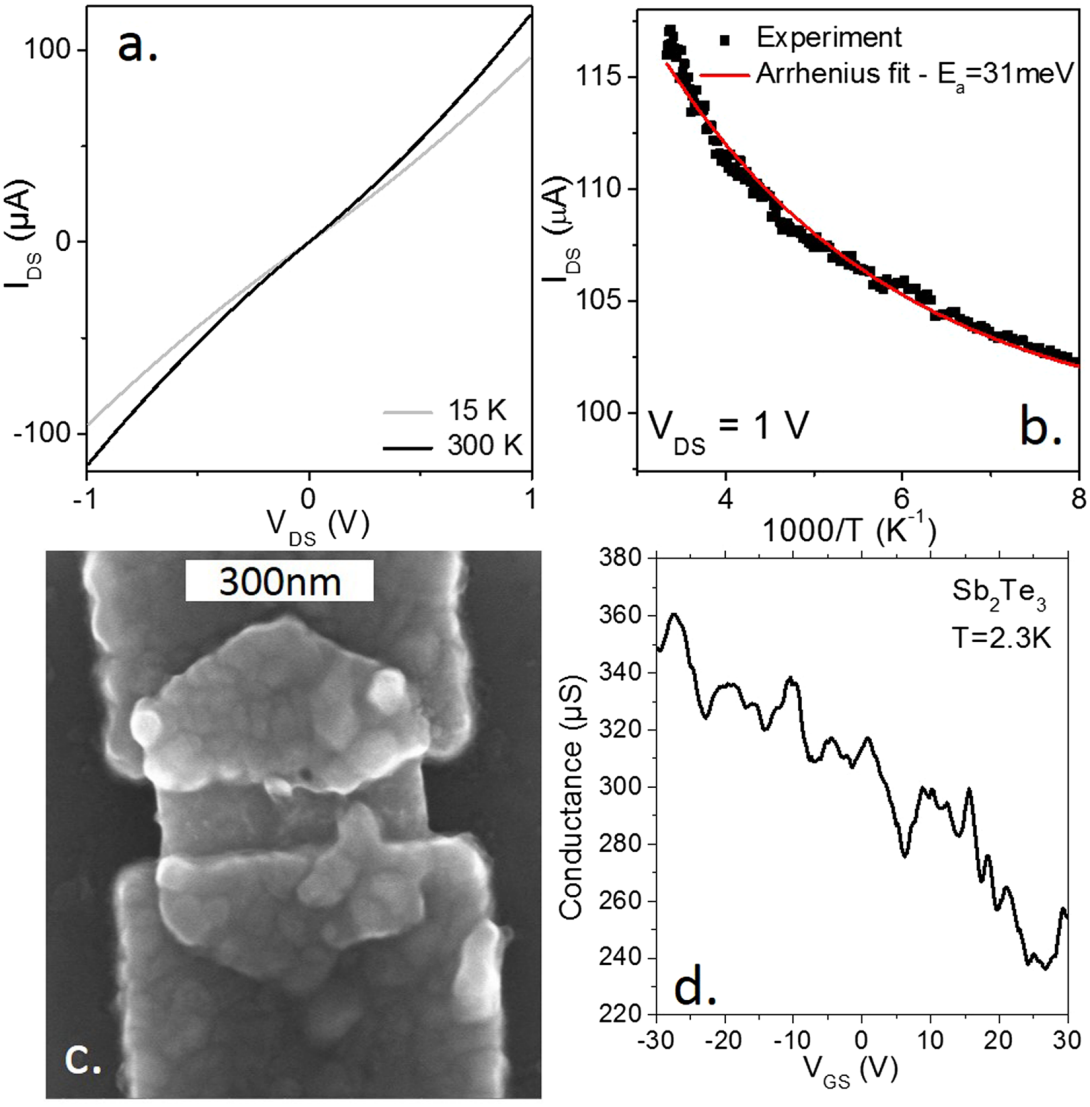
(**a**) Current as a function of applied bias for a thin film of Sb_2_Te_3_ nanoplates. The measurement is made in vacuum. (**b**) Current as a function of temperature for a thin film of Sb_2_Te_3_ nanoplates. (**c**) SEM image of a single Sb_2_Te_3_ nanoplate connected to two Al electrodes. (**d**) Transfer curve (conductance as a function of gate bias) for a single Sb_2_Te_3_ nanoplate.

In the following section, we investigate the transport properties of the single Sb_2_Te_3_ nanoplate and correlate our observations with optical method of deducing the carrier density. We first start with ensemble measurements by conducting transport on nanoplates films. The films are conductive and present an ohmic behavior at room temperature, see Fig. 2a. The temperature dependence of the current presents a small decrease of the conductance as the temperature is reduced, see Fig. 2b. between room temperature and 77 K, the temperature dependence is nicely fitted with the Arrhenius law, with a small activation energy of ≈30 meV. This is typical behaviour of thin films made of poorly coupled metallic grains^38, 39^.

Ultimately, the goal is to make single nanoparticle devices to observe the signature of surface states. In the next step, we switch from ensemble measurement to single particle measurement. Connecting a single nanocrystal can be especially difficult^40–42^, however the large lateral extension of the Sb_2_Te_3_ nanoplate makes possible the connection of a single particle using careful e-beam lithography, see Fig. 2c.

After wire-bonding of the connections to the single nanoplate, the sample is immediately cooled down to low-temperature (2.3 K). The conductance at zero drain voltage is measured with a lock-in (I_AC_ = 10 nA) as function of the gate voltage, Fig. 2d. We observe a p-type behavior with a rise of the conductance as holes (V_GS_ < 0) are injected in the nanoplate. The conductance as a function of gate voltage show reproducible fluctuations which are not simply due to electrical noise. These fluctuations are most likely related to Coulomb blockade or possibly universal conductance fluctuations^41, 42^. Indeed, while regular Coulomb peaks as a function of gate voltage are usually observed in nano-sized devices weakly coupled to the electrodes, however, when the device is more strongly coupled to electrodes as in the measurement presented in this paper, the Coulomb peaks become fainter oscillations. Furthermore, because the conductance in a nanoplatelet is not averaged on a macroscopic number of disorder configurations, the conductance fluctuates with the gate voltage because of the changing electrostatic potential responsible for electron scattering.

From the curve, we can also extract the hole mobility thanks to the relation

8 $$\mu =\frac{L}{W{C}_{\sum }{V}_{DS}}\frac{\partial i}{\partial {V}_{GS}}$$

with *L* the inter electrode spacing (≈120 nm), *W* the width of the film (≈420 nm), ${C}_{\sum }$ the sheet capacitance (11.5 nF.cm^−2^), V_DS_ the applied bias and $\frac{\partial i}{\partial {V}_{GS}}$ the transconductance. We estimate the mobility in the single plane to be in the 30–50 cm^2^V^−1^s^−1^ range which is only one decade below the typical values obtained for molecular beam epitaxy (MBE) grown film^43^.

We can then use this mobility value to estimate the transport carrier density ${n}_{trans}$. The latter relates to the conductance (G) through the relation

9 $${n}_{trans}=\frac{L}{eWt\mu }G$$

where *t* is the nanoplate thickness (≈10 nm). We estimate the value to be 1.8 × 10^19^ cm^−3^ in good agreement with our estimation based on optical measurements.

### Control of carrier density

**Figure 3:**
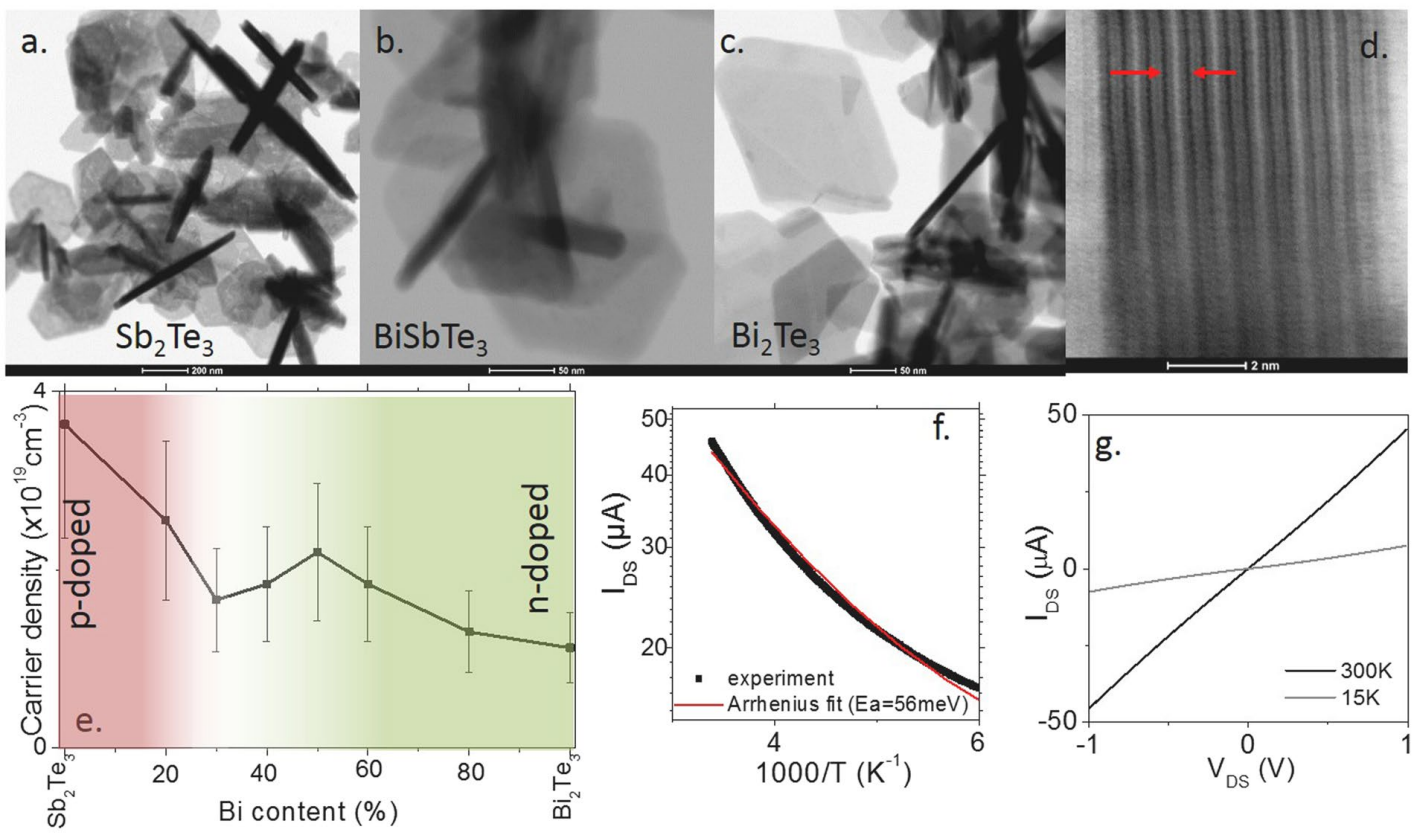
TEM images of (**a**) Sb_2_Te_3_ nanoplates, (**b**) BiSbTe_3_ nanoplates, (**c**) Bi_2_Te_3_ nanoplates. (**d**) High resolution TEM image of a Bi_2_Te_3_ nanoplate lying on the edge. (**e**) Optical carrier density as a function of Bi content in (Sb;Bi)_2_Te_3_ heterostructure nanoplates. The error bars have been obtained by repeating the measurement on several samples of a given composition. (**f**) Current as a function of temperature for a thin film of (Sb_70_;Bi_30_)_2_Te_3_ nanoplates. (**g**) Current as a function of applied bias for a thin film of (Sb_70_;Bi_30_)_2_Te_3_ nanoplates. The measurements are made in vacuum.

Transport and optical measurement agree over a bulk hole carrier density in the 2–3 × 10^19^ cm^−3^ range. We can use this value to determine the position of the Fermi level with respect to the Dirac point: ${E}_{D}-{E}_{F}$. The Fermi vector is estimated to be

10 $${k}_{F}={(3{\pi }^{2}n)}^{1/3}=0.9n{m}^{-1}$$

we can thus estimate

11 $${E}_{D}-{E}_{F}=\hslash {v}_{F}{k}_{F}=295meV$$

using ${v}_{F}$ the Fermi velocity^7^ taken as 5 × 10^5^ m.s^−1^. To reduce this energy shift between the Fermi level and the Dirac point of this material we then investigate the mixing of the *p*-type Sb_2_Te_3_ nanoplate with *n*-type Bi_2_Te_3_ material. To do so, we conduct the same reaction as before and replace a part of the antimony oleate by bismuth oleate. The reaction leads to the formation of (Sb;Bi)_2_Te_3_ heterostructured nanoplates, see Fig. 3a–c. Their lateral extension is reduced as the Bi content rises. Typically, 500 nm nanoplates are obtained for Sb rich material, while nanoplates with lateral extension below 200 nm are obtained with Bi rich condition. The lamellar aspect of the material is highlighted by conducting high resolution TEM on nanoplates lying on the side, see Fig. 3d.

We synthetize a series of (Sb;Bi)_2_Te_3_ nanoplates with various Bi content and then use the same fitting approach as for the reflectance of the film of Sb_2_Te_3_ to determine for each Bi ratio the value of the plasma frequency and the associated carrier density, see Figs 3e and S11, Table S3. As the Bi content is increased, we observe that the carrier density drops and passes by minimum around 30%. Here the carrier density is reduced by a factor 2.5 compared to pure Sb_2_Te_3_ nanoplates. The dependence of the carrier density with the Bi content is not simply a V shape curve as it may have been expected while switching from a p-type to n-type material. This results because of combination of carrier density change and effective mass change while estimating the carrier density from the plasmon frequency. We further confirm the reduction of the metallic character by measuring the transport properties of the film, see Fig. 3f,g. (Sb;Bi)_2_Te_3_ nanoplates present a stronger temperature dependence with a drop of the conductance by a factor 10 between 300 K and 15 K, while the drop was only of 20% for pure Sb_2_Te_3_ nanoplates in the same range of temperature. The high temperature activation energy extracted from the Arrhenius fit is typically twice larger and equal to 56 meV (compared to 30 meV for Sb_2_Te_3_).

**Figure 4:**
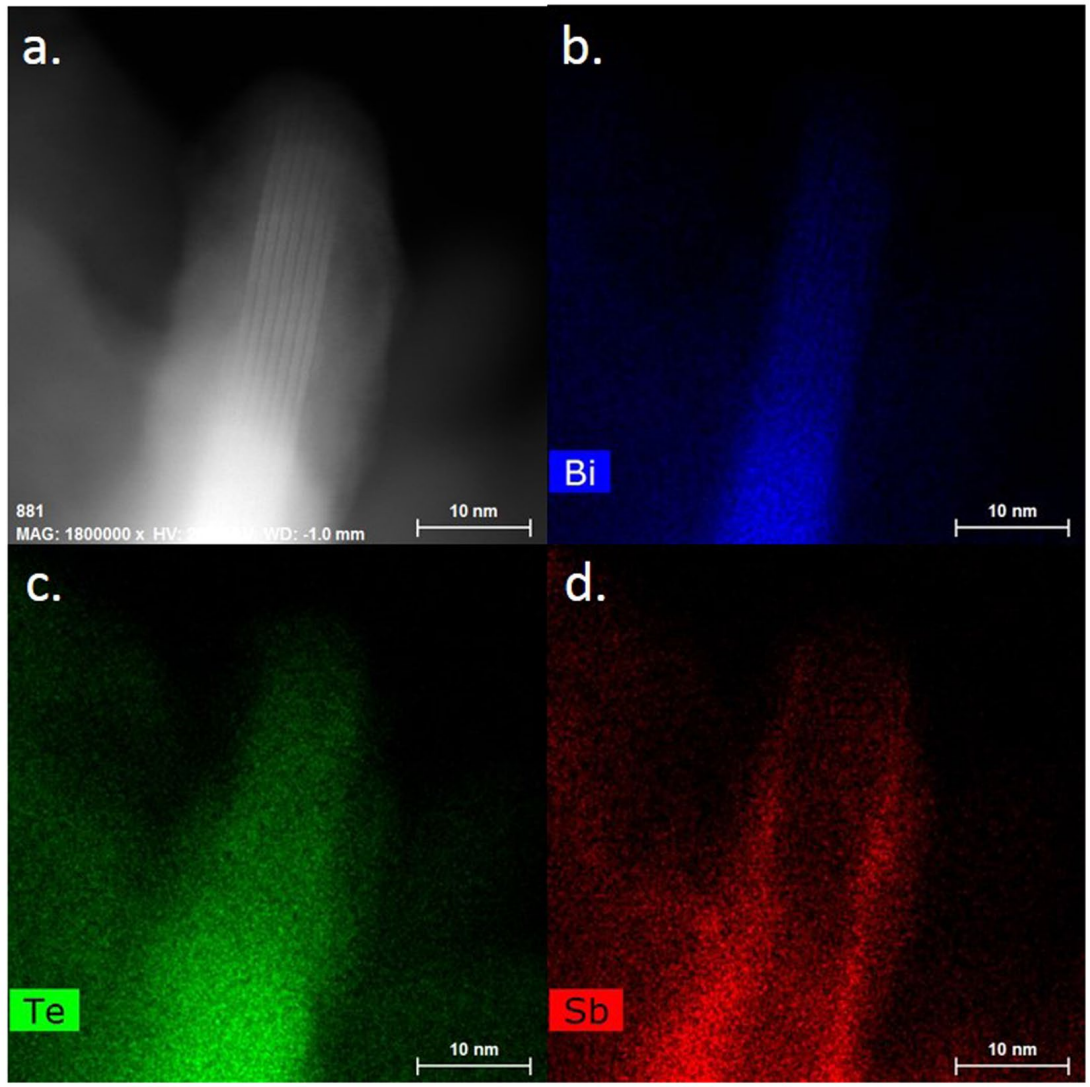
(**a**) HAADF STEM image of the (Bi,Sb)_2_Te_3_ nanoplate. The composition map of the Bi, Te and Sb of the same area are shown as separate images on part (**b**–**d**) respectively.

To unveil the exact nature of the formed (Sb;Bi)_2_Te_3_ nanoplates we use scanning transmission electron microscopy coupled with X-ray energy dispersive spectroscopy to combine nm scale resolution with chemistry composition, see Figs 4, S12 and S13. The (Sb;Bi)_2_Te_3_ nanoplates are actually not forming an homogeneous alloy, but rather from a core shell structure. The core is made of Bi_2_Te_3,_ while the shell is made of Sb_2_Te_3_. This suggest a higher reactivity of the bismuth compared to antimony towards tellurium, and is consistent with our observation that for similar growth conditions smaller nanoplates of Bi_2_Te_3_ are formed_._ Bi is more reactive towards Te which favors the nucleation step and leads to the formation of lots of small seeds. Sb_,_ which is less reactive than Bi will react immediatelty with left over Te and grow a shell on the Bi_2_Te_3_ nanoplate_,_ which behave as nucleation center. The doping control which has been demonstrated here thus differs from the approach developed for bulk or thin film in this way that charge compensation occurs at the atomic scale in the heterostructure.

## Conclusion

In this paper, we investigate the optical and transport properties of Sb_2_Te_3_ nanoplates with in mind their possible use as nanosize topological insulator material. Both reflectance and transport measurement agree on the metallic character of these objects with a carrier density in the 2–3 × 10^19^ cm^−3^ range. We then demonstrate the feasibility to conduct transport at the single particle level and determine the mobility to be between 30 and 50 cm^2^V^−1^s^−1^. Finally we demonstrate that building an heterostructure of (Sb;Bi)_2_Te_3_ with a core shell structure can be reliably used to tune the carrier density by a factor 2.5, down to the low 10^19^ cm^−3^ range.

## Experimental Section

### Chemicals

Antimony acetate (Sb(OAc)_3_, 99.99% metal basis, Aldrich), bismuth acetate (Bi(OAc)_3_, 99.999% metal basis, Aldrich), selenium powder (99.99% Strem Chemicals), tellurium powder (99.997% (trace metals basis), Alfa Aesar), Na_2_S nonahydrate (99,99%, Aldrich), Oleic acid (90% technical grade, Aldrich), Trioctylphosphine (TOP 380, 98%, Cytec), octadecene (ODE, 90% technical grade, Aldrich), hexane (95% RPE-ACS, Carlo Erba), ethanol (anhydrous 99.9%, Carlo Erba), N-methyl formamide (NMFA, 99%, Alfa Aesar.)

### Preparation of Sb(OA)_3_

In a 100 mL three neck flask, 1 g (3.35 mmol) of Sb(OAc)_3_ and 40 mL of oleic acid are loaded and put under vacuum at 85 °C for 30 min. The final solution is clear yellowish and used as a stock solution.

### Preparation of Bi(OA)_3_

In a 100 mL three neck flask, 0.5 g (1.3 mmol) of Bi(OAc)_3_ and 20 mL of oleic acid are loaded and put under vacuum at 85 °C for 30 min. The final mixture is used as a stock solution.

### Preparation of 1 M TOPTe

Trioctylphosphine complexed with tellurium is obtained by mixing 2.54 g of Te powder with 20 mL of TOP in a 50 mL three-neck flask. The solution is then degassed under vacuum for 30 min at 80 °C. The mixture is further heated under Ar at 270 °C until the powder gets fully dissolved. At this temperature the solution is orange and becomes yellow once cooled. The stock solution is kept in the glove box.

### Synthesis of Sb_2_Te_3_

In a 25 mL three neck flask, 4 mL of the antimony oleate in ODE (0.33 mmol Sb) are diluted with 10 mL of additional ODE. The flask is degassed under vacuum at 85 °C for 30 min. Then the atmosphere is switched to Ar and the temperature is raised to 200 °C. 0.5 mL of 1 M TOPTe is quickly injected and the solution rapidly turns metallic grey. The heating is continued for 5 min before the heating mantle is removed and air flow on the outside of the flask is used to cool the solution. The nanoparticles are precipitated by addition of ethanol and centrifuged for 3 min. The clear supernatant is discarded and the pellet is redispersed in hexane. The cleaning procedure is repeated two additional times.

### Synthesis of Bi_2_Te_3_

4 mL of the bismuth oleate solution (0.25 mmol Bi) and 10 mL of ODE are added to a 25 mL 3 neck flask. The flask is degassed under vacuum at 85 °C for 30 min. The atmosphere is then switched to Ar and the temperature raised to 200 °C. 0.4 mL of TOPTe (1 M) are quickly injected and the solution rapidly turns metallic grey. The heating is continued for 5 min before the reaction is cooled down. The nanoparticles are precipitated by addition of ethanol and centrifuged for 3 min. The cleaning procedure is repeated two additional times.

### Material characterization

Transmission electron microscopy (TEM) images were captured on JEOL 2010 and FEI Titan Themis microscopes. For X-ray diffraction (XRD), the nanoparticles were drop casted on a Si substrate from a hexane solution. Data was collected on a Philips X’Pert diffractometer equipped with Cu K_α_ line at 0.154 nm. Infrared spectra were measured on a Bruker Vertex 70 FTIR used in an ATR configuration with a ~700 °C globar source and a DTGS detector. The spectra were averaged 32 times with a resolution of 4 cm^−1^. Energy dispersive X-ray analysis was conducted on an Oxford probe in a FEI Magellan scanning electron microscope at 10 kV and 100 pA.

### Transport measurement

For ensemble transport measurements, we prepared, using standard lithography methods, gold electrodes on Si/SiO_2_ wafers (400 nm of oxide). The electrodes are interdigitated and include 25 pairs. Each electrodes is 2.5 mm long with a 20 µm spacing. Thin film of nanoplates over this interdigitated gold electrodes are subjected to ligand exchange with S^2−^ ions by dipping the film of nanoplatelets within a solution of Na_2_S in N-methylformamide^44^. The film is then rinsed in ethanol and dried. Measurements are made with a Keithley 2400 source-meter, using a probe station operated in air at room temperature.

For single particle measurements, the solution of Sb_2_Te_3_ nanoparticle is first drop casted on a wafer. On this wafer, the level of aggregation is high and prevents single particle connection. To obtain isolated single nanoplates, this film is transferred onto a Si/SiO_2_ wafer (300 nm of oxide) using a PDMS stamp. The film is then dipped into a 1% Na_2_S in N-methyl formamide for 45 s. Two electrodes are deposited using standard e-beam approach. Just before the metal deposition the surface is cleaned using Ar ion beam. Finally, 5 nm of titanium and 80 nm of aluminum are evaporated using an e-beam evaporator.

## Electronic supplementary material

supplementary information

## References

[1] Poudel B (2008). High-thermoelectric performance of nanostructured bismuth antimony telluride bulk alloys. Science.

[2] Talapin DV, Lee JS, Kovalenko MV, Shevchenko EV (2010). Prospects of colloidal nanocrystals for electronic and optoelectronic applications. Chem. Rev..

[3] Venkatasubramanian R, Colpitts T, Watko E, Lamvik M, El-Masry N (1997). MOCVD of Bi2Te3, Sb2Te3 and their superlattice structures for thin-film thermoelectric applications. J. Cryst. Growth.

[4] Jeon HW, Ha HP, Hyun DB, Shim JD (1991). Electrical and thermoelectrical properties of undoped Bi2Te3-Sb2Te3 and Bi2Te3-Sb2Te3-Sb2Se3 single crystals. J. Phys. Chem. Solids.

[5] Chatterjee A, Biswas K (2015). Solution-based synthesis of layered intergrowth compounds of the homologous PbmBi2nTe3n+m series as nanosheets. Angew. Chem. Int. Ed..

[6] Ando Y (2013). Topological Insulator Materials. J. Phys. Soc. Jpn..

[7] Zhang H (2009). Topological insulators in Bi2Se3, Bi2Te3 and Sb2Te3 with a single Dirac cone on the surface. Nat. Phys.

[8] Hasan MZ, Kane CL (2010). Topological insulators. Rev. Mod. Phys..

[9] Wei Z, Rui Y, Hai-Jun Z, Xi D, Zhong F (2010). First-principles studies of the three-dimensional strong topological insulators Bi2Te3, Bi2Se3 and Sb2Te3. New J. Phys..

[10] Miller GR, Li C (1965). Y. Evidence for the existence of antistructure defects in bismuth telluride by density measurements. J. Phys. Chem. Solids.

[11] Horak J, Cermak K, Koudelka L (1986). Energy formation of antisite defects in doped Sb2Te3 and Bi2Te3 crystals. J. Phys. Chem. Solids.

[12] Drasar C, Lostak P, Uher C (2010). Doping and defect structure of tetradymite-type crystals. J. Electron. Mater..

[13] Horak J, Drasar C, Novotny R, Karamazov S, Lostak P (1995). Non-stoichiometry of the crystal lattice of antimony telluride. Phys. Status Solidi A.

[14] Mott NF (1968). Metal-insulator transition. Rev. Mod. Phys..

[15] Yang F (2014). Top gating of epitaxial (Bi1-xSbx)2Te3 topological insulator thin films. Appl. Phys. Lett.

[16] He X (2012). Highly tunable electron transport in epitaxial topological insulator (Bi1- xSbx)2Te3 thin films. Appl. Phys. Lett..

[17] Hong SS, Cha JJ, Kong D, Cui Y (2012). Ultra-low carrier concentration and surface-dominant transport in antimony-doped Bi_2_Se_3_ topological insulator nanoribbons. Nat. Commun..

[18] Nasilowski M, Mahler B, Lhuillier E, Ithurria S, Dubertret B (2016). Two-dimensional colloidal nanocrystals. Chem. Rev..

[19] Saha S, Banik A, Biswas K (2016). Few-layer nanosheets of n-Type SnSe2. Chem. Eur.J.

[20] Vidal F (2013). Photon energy dependence of circular dichroism in angle-resolved photoemission spectroscopy of Bi2Se3 Dirac states. Phys. Rev. B.

[21] Lee J, Brittman S, Yu D, Park H (2008). Vapor-liquid-solid and vapor-solid growth of phase-change Sb2Te3 nanowires and Sb2Te3/GeTe nanowire heterostructures. J. Am. Chem. Soc..

[22] Teweldebrhan D, Goyal V, Balandin AA (2010). Exfoliation and characterization of bismuth telluride atomic quintuples and quasi-two-dimensional crystals. Nano Lett..

[23] Konstantatos G, Levina L, Tang J, Sargent EH (2008). Sensitive solution-processed Bi2S3 nanocrystalline photodetectors. Nano Lett..

[24] Scheele M (2009). Synthesis and thermoelectric characterization of Bi2Te3 nanoparticles. Adv. Funct. Mater..

[25] Wang W, Poudel B, Yang J, Wang DZ, Ren ZF (2005). High-yield synthesis of single-crystalline antimony telluride hexagonal nanoplates using a solvothermal approach. J. Am. Chem. Soc..

[26] Shi W, Zhou L, Song S, Yang J, Zhang H (2008). Hydrothermal synthesis and thermoelectric transport properties of impurity-free antimony telluride hexagonal nanoplates. Adv. Mater..

[27] Shi W, Yu J, Wang H, Zhang H (2006). Hydrothermal synthesis of single-crystalline antimony telluride nanobelts. J. Am. Chem. Soc..

[28] Zhou N (2014). Size-controlled synthesis and transport properties of Sb2Te3 nanoplates. RSC Adv.

[29] Zhou B, Ji Y, Yang Y, F. Li X, H. Zhu JJ (2008). Rapid microwave-assisted synthesis of single-crystalline Sb2Te3 hexagonal nanoplates. Cryst. Growth Des..

[30] Yang HQ (2015). Facile surfactant-assisted reflux method for the synthesis of single-crystalline Sb2Te3 nanostructures with enhanced thermoelectric performance. ACS Appl. Mater. Interfaces.

[31] Fei F (2015). Solvothermal synthesis of lateral heterojunction Sb2Te3/Bi2Te3 nanoplates. Nano Lett..

[32] Zhao Y, Burda C (2009). Chemical synthesis of Bi(0.5)Sb(1.5)Te3 nanocrystals and their surface oxidation properties. ACS Appl. Mater. Interfaces.

[33] Gupta G, Kim J (2013). Facile synthesis of hexagonal Sb2Te3 nanoplates using Ph2SbTeR (R = Et, Ph) single source precursors. Dalton Trans..

[34] Garje SS (2006). A new route to antimony telluride nanoplates from a single-source precursor. J. Am. Chem. Soc..

[35] Schulz S (2012). Synthesis of hexagonal Sb2Te3 nanoplates by thermal decomposition of the single-source precursor (Et2Sb)2Te3. Chem. Mat.

[36] Stepanov NP, Kalashnikov AA, Ulashkevich YuV (2010). Optical functions of Bi2Te3-Sb2Te3 solid solutions in the range of plasmon excitation and interband transitions. Opt. Spectrosc..

[37] Lucovsky G, White RM, Benda JA, Revelli JF (1973). Infrared-reflectance spectra of layered Group-IV and Group-VI transition-metal dichalcogenides. Phys. Rev. B.

[38] Moreira H (2011). Electron cotunneling transport in gold nanocrystal arrays. Phys. Rev. Lett..

[39] Tran TB (2005). Multiple cotunneling in large quantum dot arrays. Phys. Rev. Lett..

[40] Kuemmeth F, Bolotin KI, Shi SF, Ralph DC (2008). Measurement of discrete energy-level spectra in individual chemically-synthesized gold nanoparticles. Nano Lett..

[41] Wang H (2015). Effects of electron-phonon interactions on the electron tunneling spectrum of PbS quantum dots. Phys. Rev. B.

[42] Wang H (2017). Transport in a single self-doped nanocrystal. ACS Nano.

[43] Kim Y (2002). Structural and thermoelectric transport properties of thin films grown by molecular beam epitaxy. J. Appl. Phys..

[44] Lhuillier E, H. Liu Guyot-Sionnest, P. Heng L (2012). A mirage study of CdSe colloidal quantum dot films, Urbach tail, and surface states. J. Chem. Phys..

