# Supplementary material for: Electronic properties of (Sb;Bi)2Te3 colloidal heterostructured nanoplates down to the single particle level
Source: Sci Rep. 2017 Aug 29;7:9647. doi: 10.1038/s41598-017-09903-w (PMC5575357; doi:10.1038/s41598-017-09903-w)
Supplement: Supplementary file 1 — supplementary information [file 41598_2017_9903_MOESM1_ESM.pdf]

Supplementary information for

Electronic properties of $(Sb;Bi)_2Te_3$ colloidal heterostructured nanoplates down to the single particle level

Wasim J. Mir[1,2, $], Alexandre Assouline[3,$] Clément Livache[1,3], Bertille Martinez[1], Nicolas Goubet[1,3], Xiang Zhen Xu[3], Gilles Patriarche[4], Sandrine Ithurria[3], Hervé Aubin[3], Emmanuel Lhuillier[1*]

[1]Sorbonne Universités, UPMC Univ. Paris 06, CNRS-UMR 7588, Institut des NanoSciences de Paris, 4 place Jussieu, 75005 Paris, France

[2]Department of Chemistry, Indian Institute of Science Education and Research (IISER), Pune 411008, India

[3] Laboratoire de Physique et d'Étude des Matériaux, PSL Research University, CNRS UMR 8213, Sorbonne Universités UPMC Univ Paris 06, ESPCI ParisTech, 10 rue Vauquelin, 75005 Paris, France

[4] Laboratoire de Photonique et de Nanostructures (CNRS- LPN), Route de Nozay, 91460 Marcoussis, France

Table of content

1. Synthesis of Sb_2Te_3 2

1.1. Effect of temperature 2

1.2. Effect of synthesis duration 4

1.3. Effect of stoichiometry 5

1.4. Energy disperisive X-ray Spectroscopy 6

1.5. AFM image of Sb_2Te_3 8

1.6. High resolution TEM images of Sb2Te3 nanoplates 8

1. Synthesis of Bi_2Te_3 10

2. Synthesis of Bi_2Te_3/Sb_2Te_3 core/shell structure 11

1. Synthesis of Sb_2Te_3

In this section we investigate the influence of parameters such as synthesis duration, temperature and stoichiometry on the growth of the Sb_2Te_3 nanoplates.

1.1. Effect of temperature

We performed a series of syntheses where the temperature of the injection of the Te precursor was varied from 100°C to 250°C in 50°C steps. The duration of the reaction was kept constant at 5 min and the precursors were introduced in stoichiometric ratio. The most striking finding is that below 150°C, no plates are formed, see Figure S 1a. Above 150°C, Sb_2Te_3 is consistently synthesized, leading to nanoplates up to 200°C, and more sheet like material at 250°C.

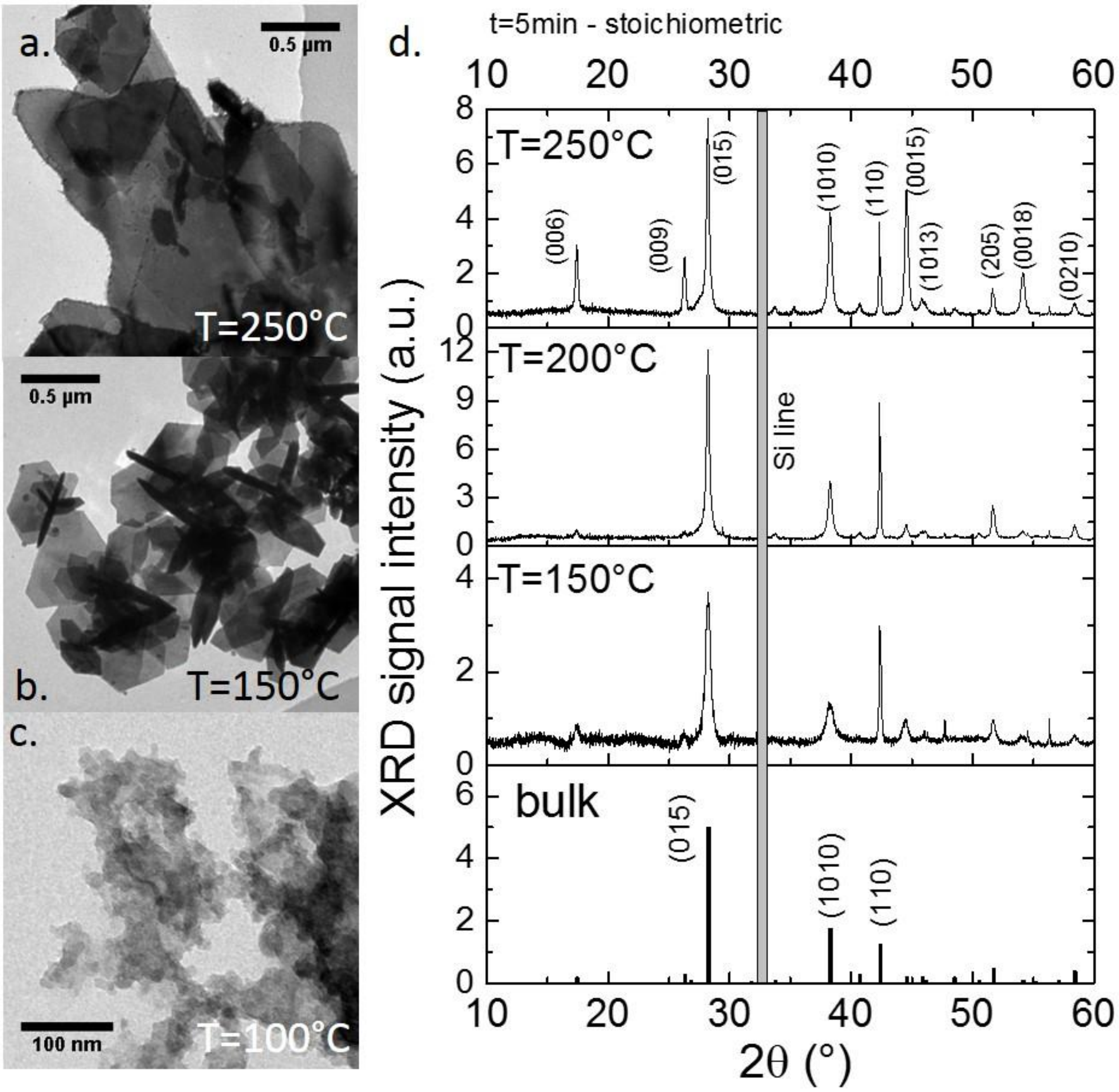

Figure S 1. a,b,c TEM images from Sb_2Te_3 nanoplates prepared at different reaction temperatures. d. XRD of Sb_2Te_3 nanoplate films prepared at different reaction synthesis temperatures. For this data the duration of the reaction was 5 min and the Sb and Te compounds were introduced in stoichiometric ratio.

The increase of the crystal size is also observed through the narrowing of the XRD peaks as the reaction temperature is increased, as seen in Figure S 1d. For all reaction synthesis temperatures, we observe a narrow peak relative to the (110) direction which suggest that this direction belongs to the plane of the nanoplate. The peaks indexed to directions perpendicular to the plate surface, narrow with increasing reaction temperature, see Figure S 2. Two phenomena contribute to this effect: (*i*) an increase in the nanoplate thickness, and (*ii*) an increase in their lateral dimension. With higher reaction temperature, larger sheets are formed. The sheets are more likely to lay flat on the substrate, increasing the relative intensity of the (00l) diffraction peaks, see for example the (006) peak at 2θ=17.4°.

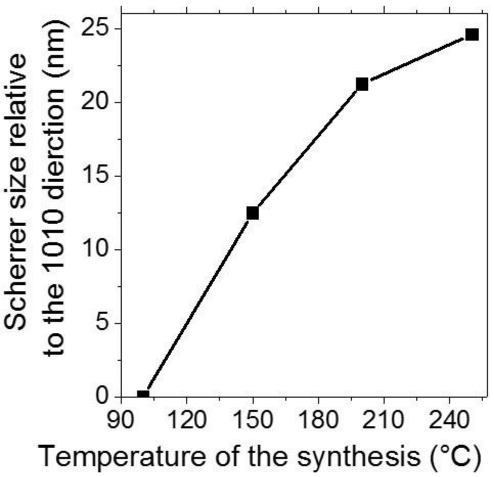

Figure S 2. Scherrer size extracted from the XRD peak in the (1010) direction as a function of temperature of the synthesis.

1.2. Effect of synthesis duration

The effect of the synthesis duration is highlighted in Figure S 3. The nanoplates get formed from the early stages of the reaction (< 1 min), see Figure S 3a. The XRD pattern changes only slightly with reaction time, and no obvious narrowing of the peaks is observed, see Figure S 3d. This suggest that a very limited further growth of the material occurs with time. This is consistent with TEM images, see Figure S 3a-c. From this observation we can conclude that most of the reaction occurs during the first minutes of the synthesis.

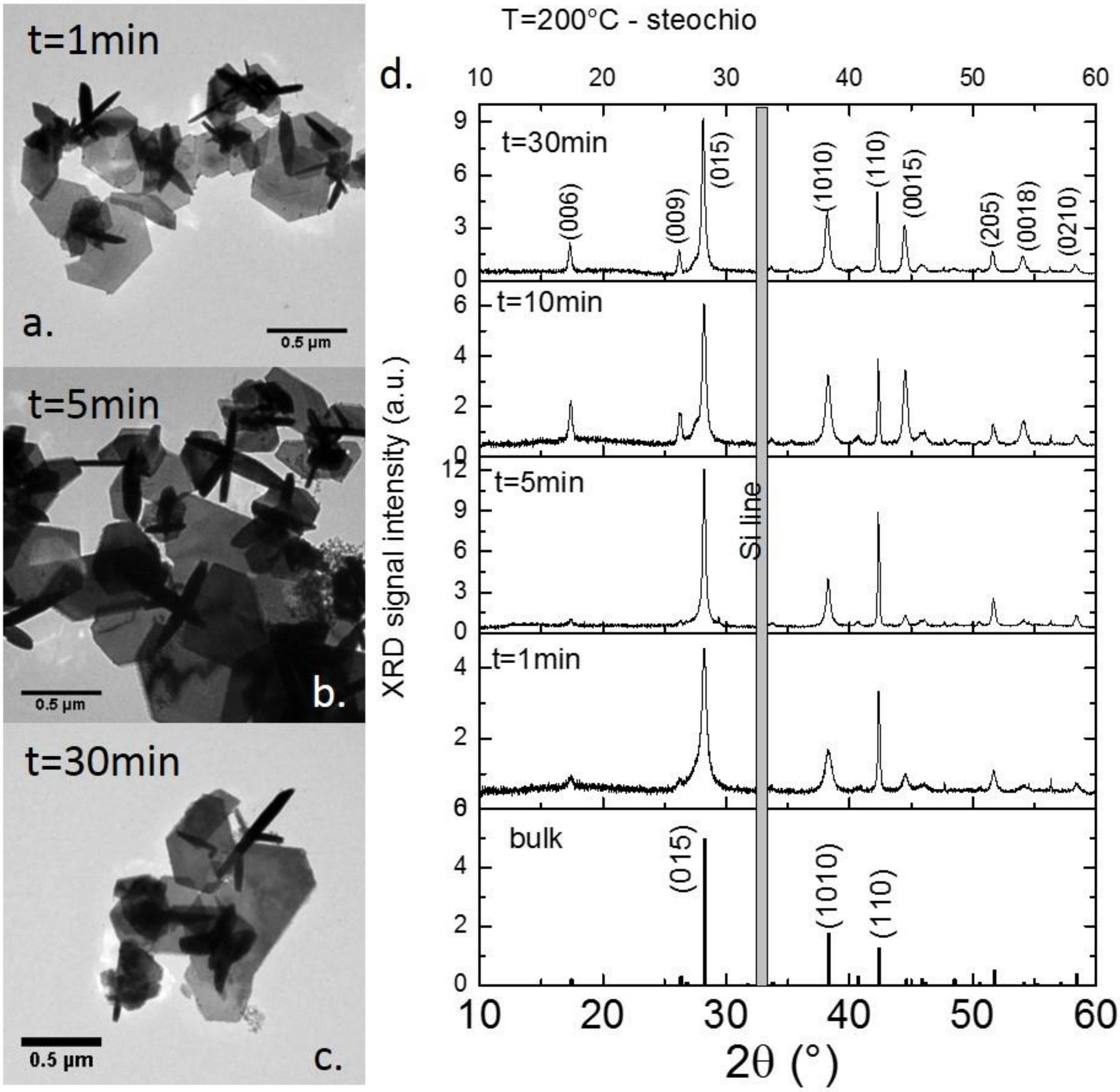

Figure S 3. a,b,c TEM images from Sb_2Te_3 nanoplates after different reaction times. d. XRD spectra of Sb_2Te_3 nanoplate films at different time of the reaction. The diffractogram from the bulk has been added for comparison. For these data the temperature of reaction have been set at 200°C while the Sb and Te compounds are introduced in stoichiometric ratio.

1.3. Effect of stoichiometry

Changing precursor stoichiometry had greater impact on the final product. With 2:3 Sb:Te or excess Sb, the XRD pattern shows only peaks consistent with Sb_2Te_3 as seen in Figure S 4d. With excess Sb, particles are observed in TEM images on the edges of the plates, as shown in Figure S 4c. With an excess of Te, diffraction peaks relative to Te (2θ = 27.54°, Figure S 4d), are observed in addition to the one from Sb_2Te_3 and are better highlighted on Figure S 5

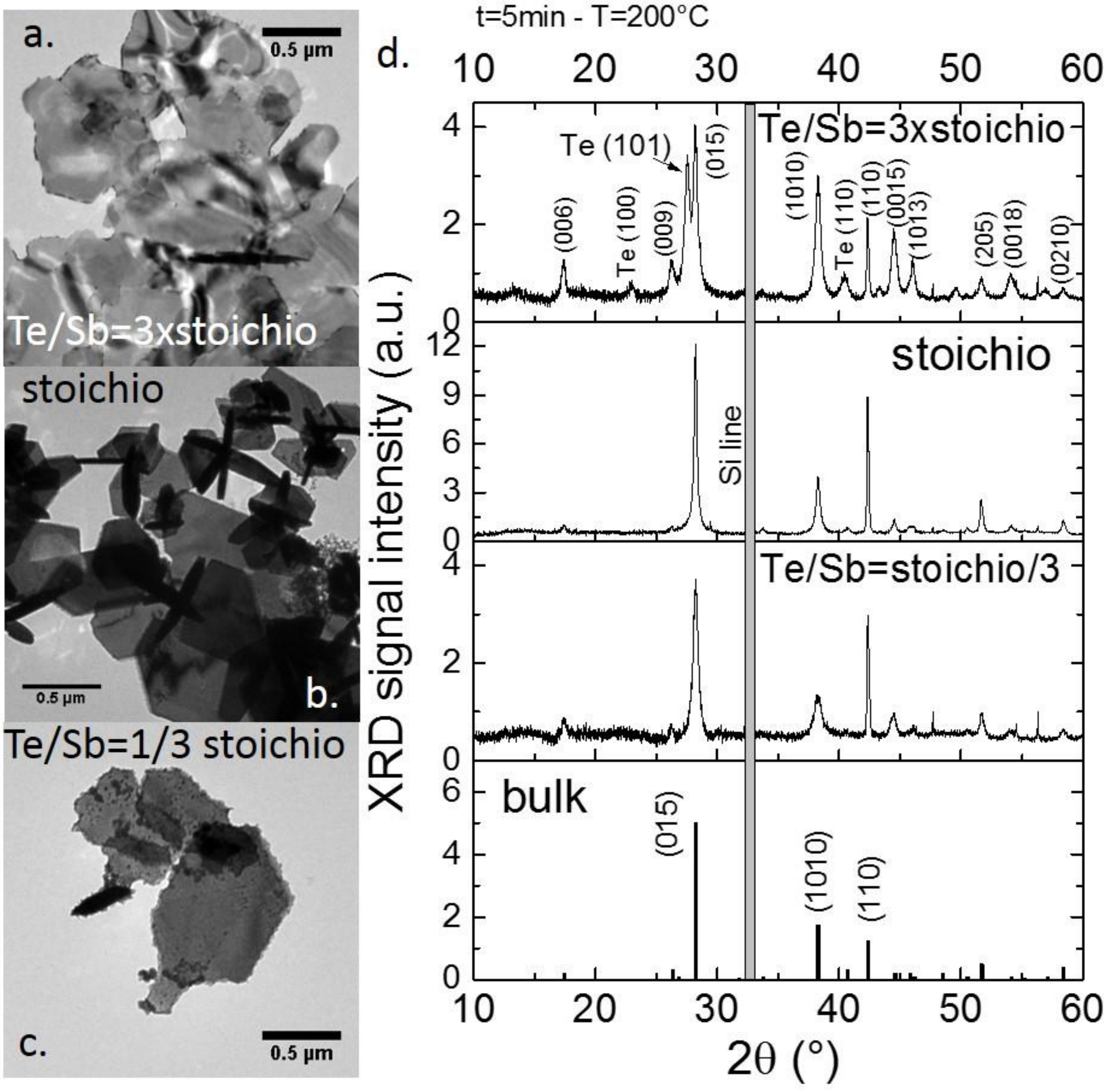

Figure S 4. a,b,c TEM images from Sb_2Te_3 nanoplates prepared at different ratio of Sb and Te precursors. d. XRD of Sb_2Te_3 nanoplate films prepared at different ratio of Sb and Te compound. The diffractogram from the bulk has been added for comparison. For this data the reaction temperature was kept at 200°C and the reaction time set at 5 min.

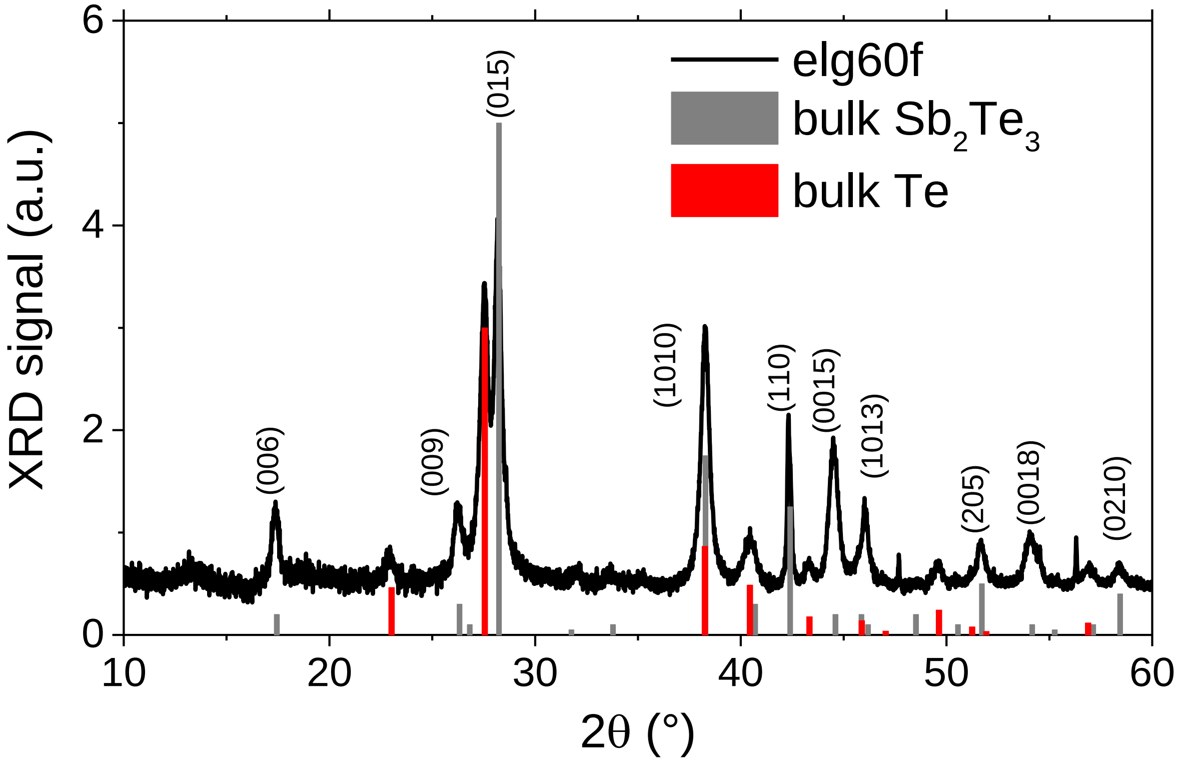

Figure S 5. XRD diffractogram from a film of Sb_2Te_3 nanoplates prepared while the Tellurium is introduced in a 3 times excess compared to stoichiometry.

1.4. Energy disperisive X-ray Spectroscopy

Energy dispersive X-ray Spectroscopy reveals the presence of Sb and Te in the formed material, see Figure S 6. The quantitative analysis of the material reveals a stoichiometry close to the expected 2/3 value, see Table S 1, but systematically with an excess of Sb.

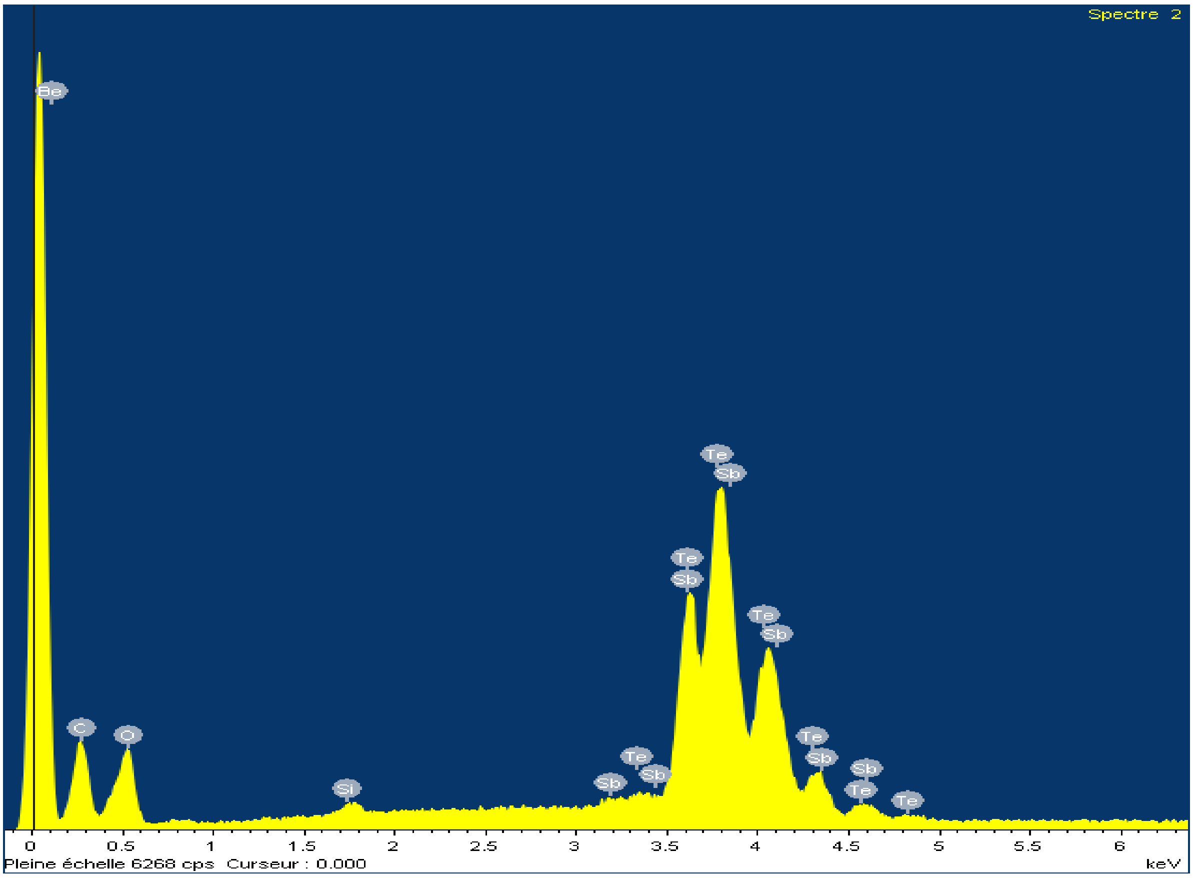

Figure S 6. Energy dispersive X ray spectrum from a film of Sb_2Te_3 nanoplates. The incident electron energy is set at 10kV.

Table S 1. EDX analysis from films of Sb_2Te_3 nanoplates

Sample	compound	O	C	Sb	Te	Cation/anion ratio
elg32	SbTe	9,9	18,3	29,55	42,27	0,70
elg32	SbTe	69,9	0	12,43	17,7	0,70
elg60b	SbTe	10,7	22,4	26,6	39,5	0,67

1.5. AFM image of Sb_2Te_3

Figure S 7 present an AFM image of a single Sb_2Te_3 nanoplate. The typical thickness is 40nm.

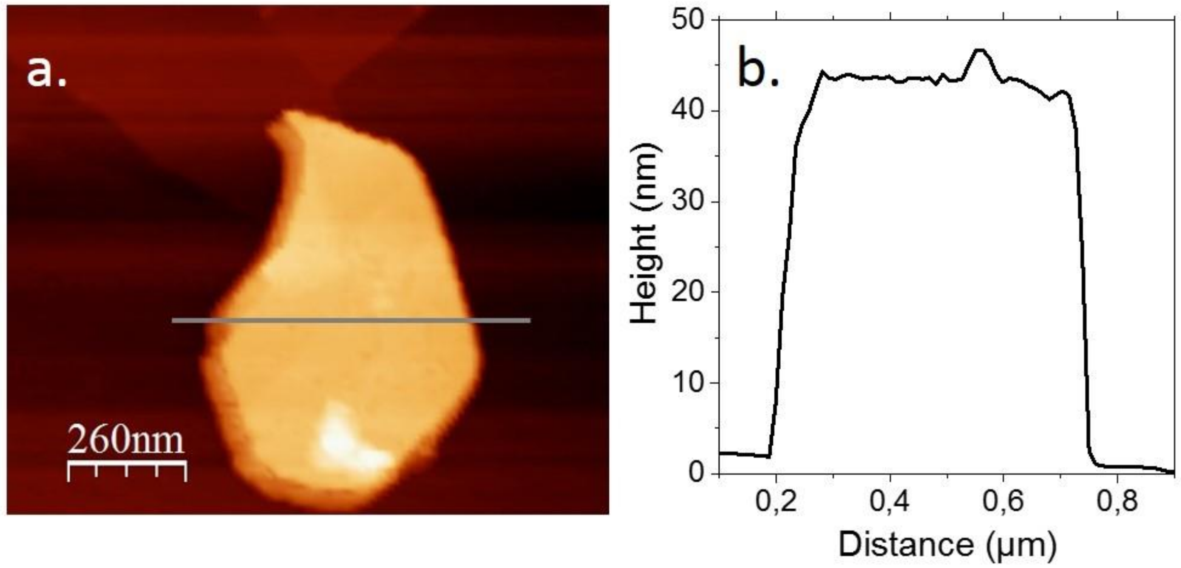

Figure S 7. a. Atomic force microscopy image of an Sb_2Te_3 nanoplate. b. height profile corresponding to the line on part a.

1.6. High resolution TEM images of Sb2Te3 nanoplates

Figure S 8 show TEM and high resolution TEM of platelets lying parallel and perpendicular to the substrate. The crystalline nature is clearly highlighted by the presence of lattice fringes.

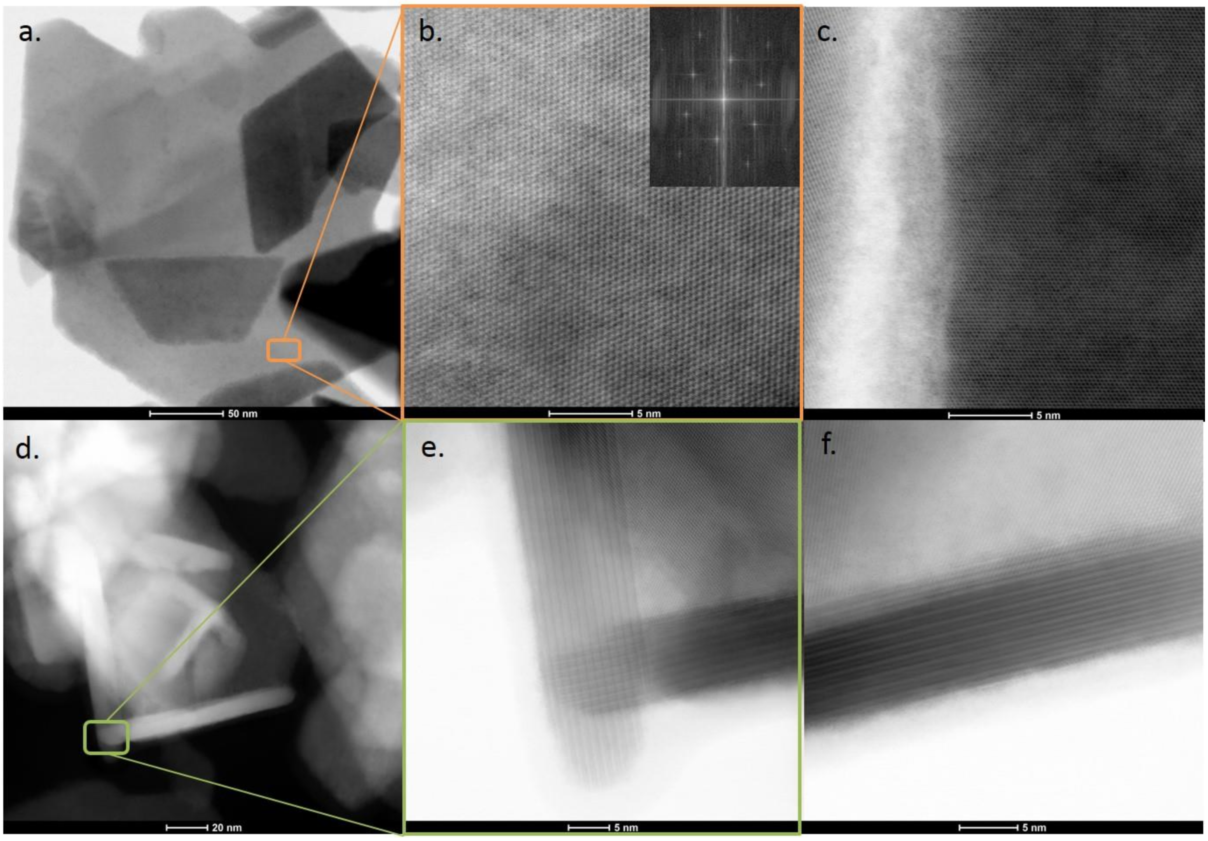

Figure S 8. a TEM image of a nanoplate lying parallel to the substrate. b. is a high resolution TEM image of a nanoplate lying parallel to the substrate. The inset is the diffraction pattern obtained from the same area. c. is a high resolution TEM image of the edge of a nanoplate lying parallel to the substrate. d. TEM image of a nanoplate lying perpendicular to the substrate. e and f are high resolution TEM images of nanoplates lying perpendicular to the substrate, which highlights the lamellar character of the Sb_2Te_3.

1. Synthesis of Bi_2Te_3

The same chemical synthesis developed for the Sb_2Te_3 can be used to obtain Bi_2Te_3 plates. The formed material presents an XRD pattern consistent with the rhombohedral phase of Bi_2Te_3, see Figure S 9. However the material is far less stoichiometric since the fomed compound include a large Te excess, see Figure S 9 and Table S 2.

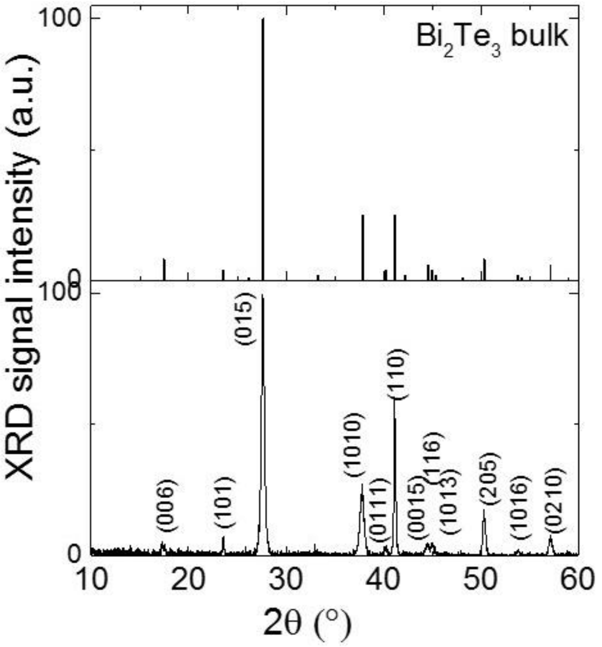

Figure S 9. XRD diffractogram from a film of Bi_2Te_3 nanoplates.

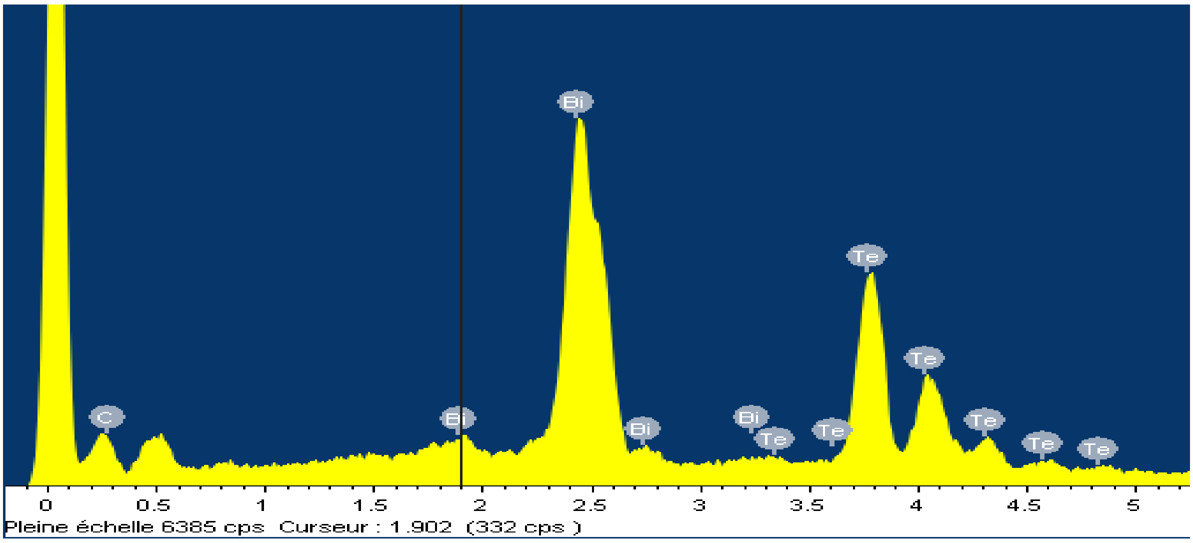

Figure S 10. Energy dispersive X ray spectrum from a film of Bi_2Te_3 nanoplates. The incident electron energy is set at 10kV.

Table S 2. EDX analysis from films of Bi_2Te_3 nanoplates

Sample	compound	O	C	Bi	Te	Cation/anion ratio
elg40	BiTe	22	0	26,7	51,3	0,52

elg40	BiTe	0	0	34,4	65,6	0,52

2. Synthesis of Bi_2Te_3/Sb_2Te_3 core/shell structure

In a 25 mL three neck flask, 2 mL of antimony oleate (0.17 mmol) + 2 mL of bismuth oleate (0.13 mmol) are introduced with 10 mL of ODE. The flask is degased under vacuum at 85 °C for 30 min. Then the atmosphere is switched to Ar and the temperature raised to 200 °C. 0.5 mL of TOPTe (1M) are quickly injected and the solution turns rapidly metallic grey. The heating is continued for 5min before the heating mantle is removed and fresh air is used to cool down the flask. The nanoparticles are precipitated by addition of ethanol and centrifuged for 3min. The clear supernatant is discarded and the formed pellet is redispersed in hexane. The cleaning procedure is repeated two other times. The Figure S 11 shows the reflectance spectra for the $(Sb;Bi)_2Te_3$ nanoplates with various Bi content. The Table S 3 provides the obtained fitted parameters.

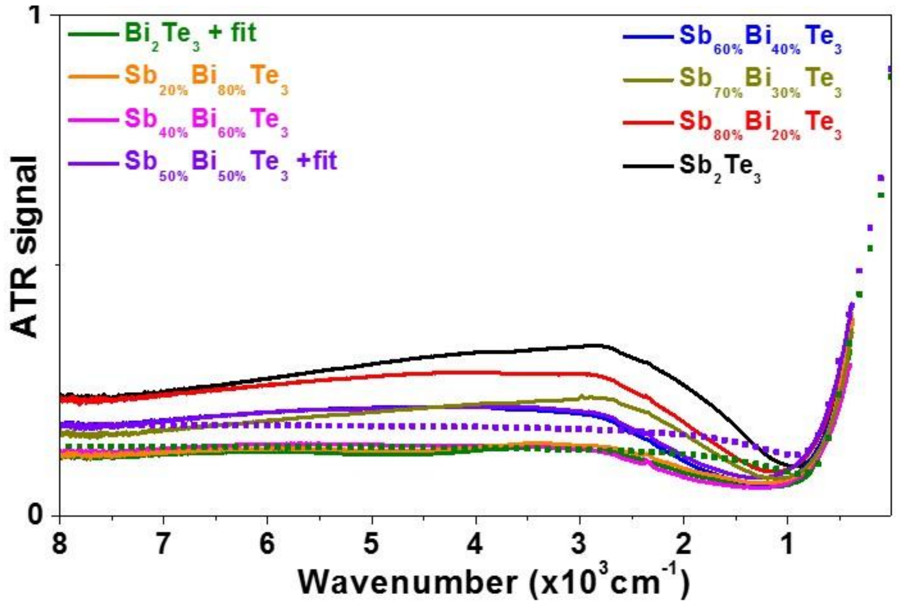

Figure S 11. ATR signal for thin film of $(Sb;Bi)_2Te_3$ nanoplates with various Bi content. The fit for the Bi_2Te_3 and $SbBiTe_3$ material have been added.

Table S 3 Values of the carrier density, dielectric constant and damping rate obtained by fitting the curve of Figure S 11.

Bi ratio	Sb_2Te_3	$(Sb_{80};Bi_{20})_2Te_3$	$(Sb_{70};Bi_{30})_2Te_3$	$(Sb_{60};Bi_{40})_2Te_3$	$(Sb_{50};Bi_{50})_2Te_3$	$(Sb_{40};Bi_{60})_2Te_3$	$(Sb_{20};Bi_{80})_2Te_3$	Bi_2Te_3
ω_p (cm^{-1})	1767	1734	1548	1629	1780	1558	1558	1447
N ($x10^{19}cm^{-3}$)	3.6	2.55	1.66	1.84	2.2	1.3	1.3	1.12
ε_∞	10.4	7.6	6.2	6.2	6.2	4.8	4.8	4.8
$1/\gamma$ (fs)	17.4	11.8	11.8	10.4	9	7.6	9	10.4

Table S 4. EDX analysis from films of $SbBiTe_3$ nanoplates

**compound**	**O**	**C**	**sb**	**Bi**	**Te**	**Cation/anion ratio**
SbBiTe	11,42	69,6	3,5	3,37	12,1	0,57
SbBiTe	12,6	61,2	4,7	4,84	16,6	0,57

HAADF STEM imaging coupled with EDX analysis reveals that the nanoplates actually have a core-shell structure with Bi_2Te_3 localized in the inner part of the nanoplate and the Sb_2Te_3 on the external part of the nanoplate, see Figure S 12 and Figure S 13.

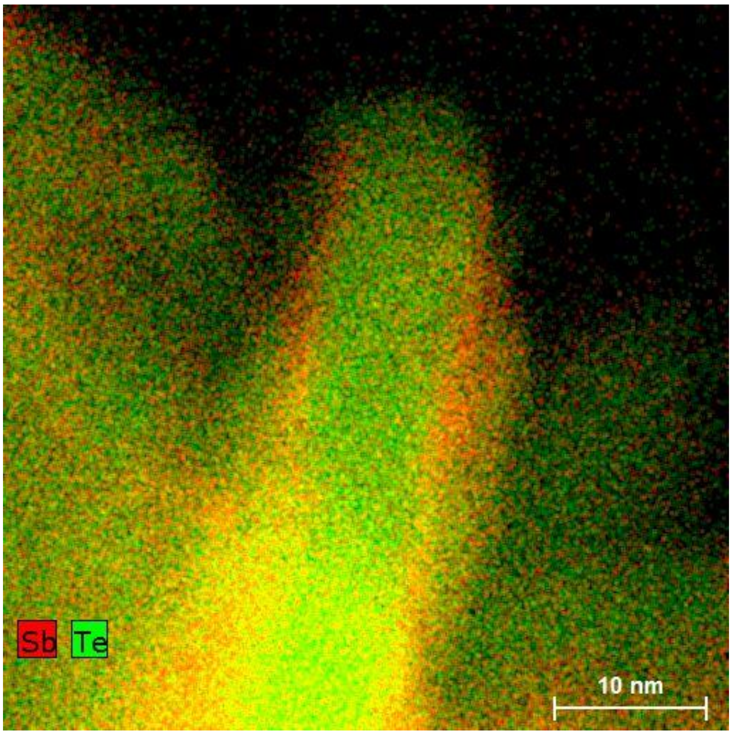

Figure S 12. Superimposed EDX mapping for Sb and Te obtained from the image of Figure 4.

The Figure S 12 shows that the Te is localized all over the platelets, while the Sb is only on the external part

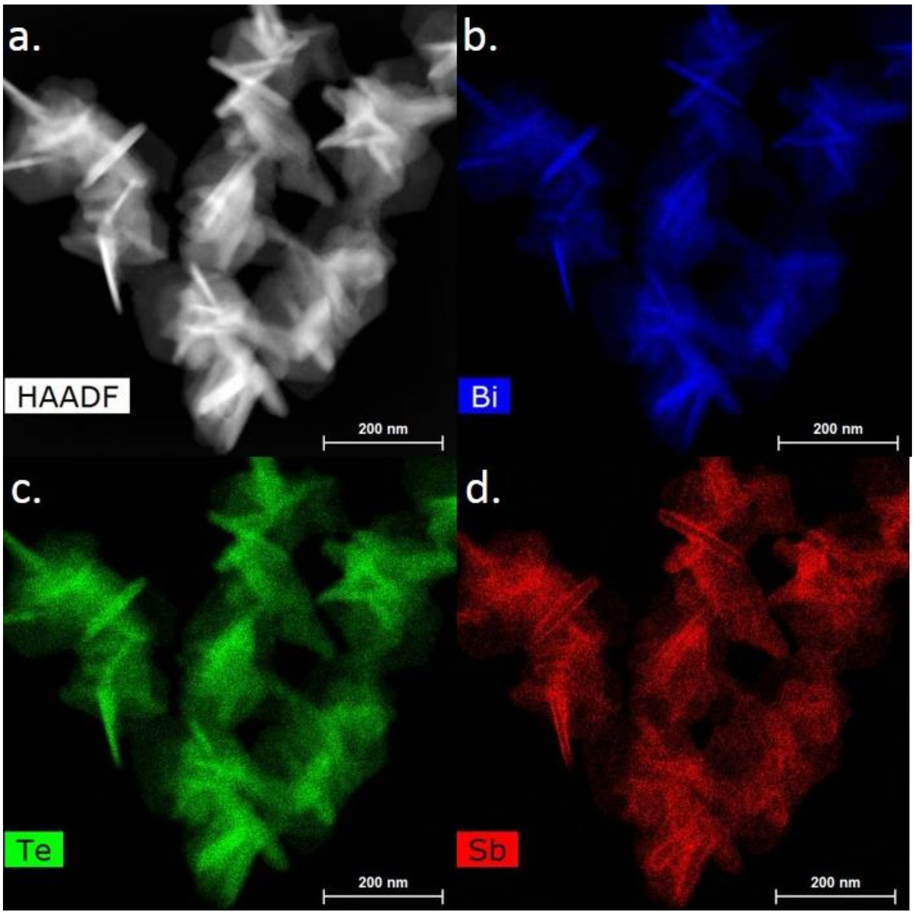

Figure S 13 a. HAADF STEM image of the (Bi;Sb)$_2$Te$_3$ nanoplates. The composition cartography of the Bi, Sb and Te of the same area are shown respectively on part b, c and d.
